# Current Insights into Diagnosis, Prevention Strategies, Treatment, Therapeutic Targets, and Challenges of Monkeypox (Mpox) Infections in Human Populations

**DOI:** 10.3390/life13010249

**Published:** 2023-01-16

**Authors:** Mitesh Patel, Mohd Adnan, Abdu Aldarhami, Abdulrahman S. Bazaid, Nizar H. Saeedi, Almohanad A. Alkayyal, Fayez M. Saleh, Ibrahim B. Awadh, Amir Saeed, Khalid Alshaghdali

**Affiliations:** 1 Department of Biotechnology, Parul Institute of Applied Sciences and Centre of Research for Development, Parul University, Vadodara 391760, India; 2 Department of Biology, College of Science, University of Ha’il, Hail P.O. Box 2440, Saudi Arabia; 3 Department of Medical Microbiology, Qunfudah Faculty of Medicine, Umm Al-Qura University, Al-Qunfudah 21961, Saudi Arabia; 4 Department of Medical Laboratory Science, College of Applied Medical Sciences, University of Hail, Hail 55476, Saudi Arabia; 5 Department of Medical Laboratory Technology, Faculty of Applied Medical Sciences, University of Tabuk, Tabuk 71491, Saudi Arabia; 6 Department of Medical Microbiology, Faculty of Medicine, University of Tabuk, Tabuk 71491, Saudi Arabia; 7 Laboratory and Blood Bank Department, Qunfudah General Hospital, Al-Qunfudah 28821, Saudi Arabia

**Keywords:** Monkeypox virus, Mpox, orthopoxvirus, zoonotic disease, infectious diseases, emerging diseases

## Abstract

In the wake of the emergence and worldwide respread of a viral infection called Monkeypox (Mpox), there is a serious threat to the health and safety of the global population. This viral infection was endemic to the western and central parts of Africa, but has recently spread out of this endemic area to various countries, including the United Kingdom (UK), Portugal, Spain, the United States of America (USA), Canada, Sweden, Belgium, Italy, Australia, Germany, France, the Netherlands, Israel, and Mexico. This is a timely review focusing on recent findings and developments in the epidemiology, clinical features, therapeutic targets, diagnosis, prevention mechanisms, research challenges and possible treatment for Mpox. To date (29 November 2022), there have been around 81,225 reported cases of Mpox. In most cases, this illness is mild; however, there is a fatality rate ranging from 1 to 10%, which might be increased due to associated complications and/or secondary infections. There is a real challenge in the diagnosis of Mpox, since its symptoms are very similar to those of other infections, including smallpox and chickenpox. Generally, to prevent/limit the risk and transmission of Mpox, the detection and isolation of infected individuals, as well as hand hygiene and cleanliness, are essential and effective approaches to control/combat this viral infection. Nevertheless, updated information about Mpox from different angles is lacking. Thus, this review provides updated and comprehensive information about the Mpox illness, which should highlight the global burden, pathogenicity, symptoms, diagnosis, prevention measures and possible treatment of this emerging disease.

## 1. Introduction

**Figure 1:**
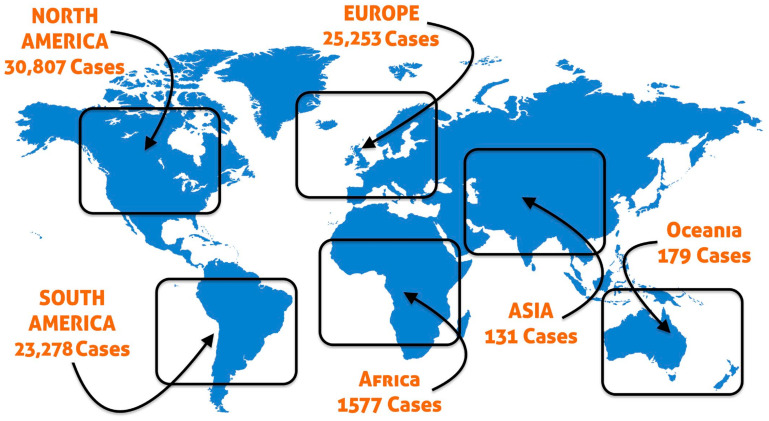
A world map showing the distribution of Monkeypox (Mpox) cases around the world based on suspected and confirmed cases as of 29 November 2022 (Source—https://www.cdc.gov/poxvirus/Mpox/response/2022/world-map.html, accessed on 30 November 2022).

Monkeypox (Mpox) virus is a double-stranded deoxyribonucleic acid (DNA) virus, causing Mpox illness in humans and other animals, that is rapidly spreading worldwide. On 29 November 2022, around 81,225 cases of Mpox were confirmed and/or suspected from about 111 countries [1] (Figure 1). In addition to the world’s continued fight against the coronavirus disease (COVID-19) pandemic, this recently emerging disease has added another global burden to worldwide health. As an added advantage, unlike COVID-19, which was detected during the early part of 2020, scientists have gained a great deal of insight into Mpox in the past few years. Nevertheless, detailed and up-to-date information about Mpox is very limited, and the future outcome of this viral infection is not extensively discussed. Therefore, this review focuses on the Mpox illness in terms of prevalence, transmission and pathogenicity, clinical manifestations, diagnostic approaches, prevention measures, and possible treatment or/and vaccination.

## 2. Survey Methodology

Various scientific search engines, such as ScienceDirect, PubMed, Scopus, Google Scholar and MEDLINE were used to search and retrieve related published papers/literature about Mpox since 1958. The keywords/phrases/sentences that were used to search for relevant papers/literatures/data related to Mpox infection in order to generate only Mpox infection-related literature were as follows: Mpox virus, epidemiology of Mpox infection, clinical characteristics of Mpox infection, diagnosis and treatment of Mpox infection, and pathogenesis of Mpox virus. Exclusion criteria were also set. Studies that did not meet the current inclusion criteria or were irrelevant to the topic, abstracts, conference proceedings, editorials, and commentaries with insufficient data were excluded.

## 3. History

The Mpox virus, which is a member of the Orthopoxvirus family, causes a zoonotic disease similar to smallpox in humans. The variola virus (the cause of smallpox), the vaccinia virus, and the cowpox virus are all members of this genus [2,3,4]. They are 200 to 250 nm brick-shaped enveloped viruses with characteristic surface tubules and a dumb-bell-shaped core component [5]. In the period between 1958 to 1968, a large number of primates were imported from Asia, and a few from Africa, into Europe and the United States to be used in developing and testing the polio vaccine [6]. While they were in transit, they were often accompanied by other wild animals, which created a lot of opportunities for infection to spread [6]. At the Statens Serum Institut, Copenhagen, Denmark, Preben von Magnus first noticed Mpox in 1958, when he observed two non-fatal outbreaks of Mpox affecting laboratory cynomolgus monkeys after 51 and 62 days of the shipments from Singapore via air transport [7,8]. Subsequently, while performing routine investigations, it was found that the Mpox virus was present in the kidneys of monkeys that showed no symptoms and had otherwise been healthy [9].

In Asia, the virus has never been found, and it is generally believed that the occurrence of the virus in Asian monkeys is a result of their catching the disease in captivity or in transit [6]. It is thought that some previous cases of a pox outbreak, thought to be smallpox, among monkeys, may have actually been Mpox [7]. In the 1960s, several lab monkeys were often found to have Mpox in European and American laboratories, including at the Walter Reed Army Institute of Research in 1962, where several monkeys had antibodies and no symptoms following exposure to Mpox [9]. After 1968, there were no more cases of monkeys dying in laboratories, as the conditions for monkeys in labs improved and the number of monkeys coming from Asia and Africa decreased [6]. Until 1965, when Mpox was first discovered at Rotterdam Zoo, it was thought that Mpox only occurred in primates [10]. However, the disease was first detected among the giant anteaters from Central and South America, before affecting several orangutans, chimpanzees, gorillas, guenons, squirrel monkeys, macaques, marmosets and gibbons, and 11 of the 23 animals died as a result of it [9]. Orangutans were most severely affected by the disease [11]. At the time, the Mpox virus was also isolated from the kidneys of healthy monkeys, but it was later revealed that the virus was most likely due to contamination from samples from the Zoo that were undergoing examination at the same laboratory as the Mpox virus [6,9].

During the smallpox eradication efforts in 1970, the first documented cases of Mpox in humans were found in six unvaccinated children, with the first one being in a 9-month-old boy in the Democratic Republic of the Congo (formerly Zaire) [9,12]. Three of the others were from Liberia and Sierra Leone [9,13]. One notable fact about Mpox is that it is less contagious than smallpox [14]. In the period from 1981 to 1986, more than 300 cases of Mpox were reported in the DRC, the majority of them linked to animal contact. Approximately 88% of the cases resulting from human-to-human transmission were reported in the Democratic Republic of the Congo in 1996 [14]. In equatorial central and western Africa, there are regular outbreaks of viral infections with death rates around 10% and human-to-human transmission rates around the same [15]. There was no outbreak of Mpox in the United States until 2003, when a concurrent Mpox outbreak took place in the rain forests of western and central Africa [16].

A number of cases have been traced back to Ghanaian rodents that were imported [14]. Prairie dogs in the regions of Africa contracted the disease and passed it on to their owners [14]. Although deaths in prairie dogs were observed, no human fatality was recorded [14]. It has been reported that the disease had spread to at least ten African countries between 1970 and 2019, mainly in central and west Africa [12].

A case of Mpox was diagnosed in the United Kingdom (UK), in 2018, among two passengers who had travelled from Nigeria [17]. In the UK, the first case of human-to-human transmission outside of Africa was confirmed in that year [18]. It is possible that the person contracted the disease from contaminated bed linen, while working as a healthcare worker. After that, more cases were reported in the UK between 2019 and 2021 [18]. The disease has also been reported in travellers from Israel and Singapore [19].

## 4. Origin and Spread of Mpox Virus

Since the first human case of Mpox was reported in the Democratic Republic of the Congo, over 65 years ago, there have been various outbreaks and sporadic cases in many areas of central and west Africa. It has been reported that most cases have occurred in remote rainforest areas of the Congo Basin, particularly in the Democratic Republic of the Congo. Human cases have also increasingly been reported in parts of central and western Africa [20].

A total of 11 African countries have reported human Mpox cases since 1970, including Benin, Cameroon, the Central African Republic, the Democratic Republic of the Congo, Gabon, Cote d’Ivoire, Liberia, Nigeria, the Republic of the Congo, Sierra Leone and South Sudan [21]. During 1996–97, the Democratic Republic of the Congo experienced an outbreak that was characterized by a lower-case fatality ratio and a higher attack rate than normal. It was found, in this case, that chickenpox (caused by a virus other than Orthopoxvirus, called Varicella-Zoster virus) and Mpox were occurring concurrently. This could explain any observed changes in transmission dynamics in this case. The outbreak in Nigeria that began in 2017 was a large one, with over 500 suspected cases and 200 confirmed cases, and a case fatality ratio of approximately 3% [22]. Until this day, cases continue to come to light.

Since Mpox does not only affect countries in Africa, but the rest of the world as well, the disease is of global importance when it comes to public health. A Mpox outbreak occurred in the United States in 2003, the first Mpox outbreak outside of Africa, and the cause was found to be contact with infected pet prairie dogs. They were housed together with Gambian pouched rats and dormice that were imported into the country from Ghana [23]. During this outbreak, over 70 cases of Mpox were reported in the United States. Mpox has also been reported to have been transferred from travellers from Nigeria to Israel (September 2018) as well as to travellers from the UK (September 2018), United States of America (December 2019) and Singapore (May 2019). Multiple Mpox cases have been reported in several countries outside the endemic area since May 2022. Currently, there are studies being conducted to better understand the epidemiology, sources of infection, and patterns of transmission of the disease [24].

**Figure 2:**
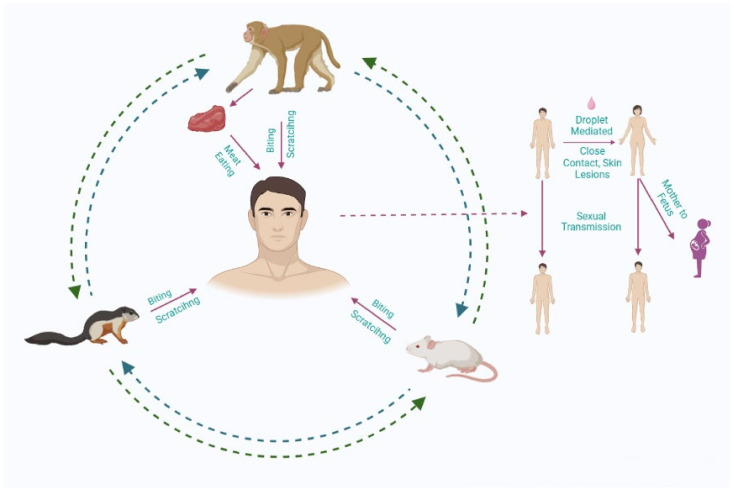
The transmission routes of the Monkeypox (Mpox) virus.

A zoonotic (animal-to-human) transmission can take place through direct contact with blood, bodily fluids or by contact with the lesions on the skin or mucosa of animals that are infected. There has been evidence of Mpox virus infection in many species of animals found in Africa, including tree squirrels, rope squirrels, Gambian pouched rats, and dormice, as well as various monkey species [2] (Figure 2). It remains unclear which animal is the natural reservoir for Mpox, but rodents would probably be the most likely. It is possible to contract the disease from eating infected meat that has not been properly cooked. In some cases, people living in or near forested areas may be indirectly exposed to infected animals due to their proximity [25].

Transmission of the virus from person-to-person can be caused by close contact with respiratory secretions, lesions on the skin of an infected person, or recently contaminated objects. Health workers, household members and other close contacts of persons who are infected with the disease are at greater risk, as droplet respiratory particles are required to transmit the infection via prolonged face-to-face contact [26]. It has been documented in the past few years that the longest ever documented chain of transmission has increased from six to nine successive person-to-person infections within one community [27]. There is also the possibility that Mpox can be transmitted via the placenta from a mother to a foetus (which can lead to congenital Mpox) [28]. The close physical contact that can occur in the context of Mpox is well known as a risk factor for transmission; as of now, it is clear that Mpox can be transmitted specifically through sexual means [29]. Therefore, it is important to conduct further research to gain a better understanding of the disease.

## 5. Transmission and Circulation of Mpox in Human Populations

Mpox first became known to humans in 1970 in Basankusu, Équateur Province, in the Democratic Republic of the Congo (formerly Zaire) [30]. The WHO recorded 338 confirmed cases between 1981 and 1986 in the Democratic Republic of the Congo/Zaire (case fatality ratio—9.8%) [31]. In 1996–1997, a second outbreak of human illness was reported in the Democratic Republic of the Congo and Zaire province [32]. Between 1991 and 1999, there were 511 cases reported in the Democratic Republic of the Congo/Zaire. The Congo Basin clade of diseases still continues to be endemic in the DRC and has a high case fatality ratio [31]. By May 2022, the case fatality rate (CFR) for outbreaks from the past had been around 3% to 6%, while the CFR for the outbreak that occurred in 2022 remained less than 1%. There was no record of human-to-human transmission of Mpox until the 2022 outbreak of Mpox in Europe [33]. In 2003, Mpox was reported in the Midwestern United States among owners of pet prairie dogs belonging to Clade II, the first outbreak outside Africa. There were reportedly 71 people infected with the disease, none of whom died as a result [33].

Mpox has traditionally been associated with tropical rainforests and their ecology. It was only in 2005 that the pattern was broken, when 49 cases of the disease were reported in South Sudan (a region that was formerly part of Sudan) with no deaths. Genetic analysis indicates that the virus did not originate in Sudan, but was imported from the Democratic Republic of the Congo [34].

A significant increase in Mpox cases has occurred in central and western Africa, especially in the Democratic Republic of the Congo, where 2000 cases per year have been reported between 2011 and 2014. There are many instances in which the data collected is incomplete or unconfirmed, causing unrealistic estimations of the number of Mpox cases over a period of time. In spite of this, it has been suggested that the number of reported cases of Mpox has increased and the geographical occurrences became more widespread in 2018 [31].

### 5.1. United States Outbreak (2003)

An infant became ill after being bitten by a prairie dog bought at a swap meet near Milwaukee, Wisconsin, in May 2003 [35]. A total of 71 cases of Mpox had been reported by the end of 2003. All of the cases have been linked to the importation of Gambian pouched rats from Accra, Ghana, in April 2003, by an exotic animal distributor in Texas. There were no reported deaths from this outbreak. A Mpox patient generally experiences prodromal symptoms, such as fever, headaches, muscle aches, chills, drenching sweats, and drenching fever, when they have Mpox. Infected individuals were found to have non-productive coughs in about a third of the cases [36].

### 5.2. Nigeria Outbreak (2017–2019)

A Mpox outbreak was reported in the south-eastern and southern parts of Nigeria. The virus spread to the following states: AkwaIbom, Abia, Bayelsa, Benue, Cross River, Delta, Edo, Ekiti, Enugu, Imo, Lagos, Nasarawa, Oyo, Plateau, Rivers and Federal Capital Territory [37,38]. According to the information provided by the Nigeria Center for Disease Control, a total of ten cases of Mpox in humans were reported during the period of 1971 to 1978 [39]. After almost 39 years, the outbreak started again in September 2017, and it has continued across multiple states as of May 2019 [40,41]. In 2017, the first case of Mpox in humans was reported from the state Bayelsa. The outbreak was characterized by infection, predominantly among young, male adults, without significant transmission to others, which is different from previous reports of the west African clade (Clade is a a group of biological taxa (such as species) that includes all descendants of one common ancestor). Several cases of genital ulcers, syphilis and HIV co-infection, as reported by the Niger Delta University Teaching Hospital, were found in young adults [42]. There have been reports from the US Center for Disease Control and Prevention that American travellers who have returned from Lagos and Ibadan have contracted Mpox [43].

### 5.3. United Kingdom Cases (2018)

There was a case of Mpox reported in the UK for the first time in September 2018. It is believed that the person, a Nigerian citizen, contracted Mpox in Nigeria before travelling to the UK [44]. In accordance with Public Health England, the individual had been staying at a naval base in Cornwall when he was moved to the Royal Free Hospital’s unit for infectious diseases. The people who had been in contact with the patient since he contracted the disease were contacted [45]. There was a second case in the town of Blackpool, England [44], along with yet another case that was associated with a medical staff member treating the Blackpool case. A fourth Mpox case was reported in England on 3 December 2019, when a person in southwest England was diagnosed with the disease. This individual had travelled from Nigeria to the UK [46]. A large outbreak of Mpox has been reported in the UK in 2022 as part of the larger epidemic of Mpox caused by the west African clade of the Mpox virus. A total of 3504 cases of Mpox have been confirmed as of 3 October 2022, and 150 cases are highly probable [47,48].

### 5.4. Singapore Case (2019)

It was reported on 8 May 2019 that a 38-year-old man from Nigeria, who had travelled to Singapore from Nigeria, was hospitalized in an isolation ward at the National Centre for Infectious Diseases in Singapore after it was confirmed that he was the first Mpox case in Singapore. This resulted in the quarantining of 22 individuals. The case may have been connected to an outbreak that was occurring concurrently in Nigeria [49].

### 5.5. 2021 Cases

As of the 24th of May, Public Health Wales had identified three cases of Mpox from a single household in the UK. Health Secretary Matt Hancock announced the cases in an address to the House of Commons. It was noted that the person with the infected organism had travelled from Nigeria. It was reported that a second case occurred on 2nd June, and a third case was reported on 24th June [46]. There was a case of Mpox in the US on the 14th of July after an American returned from Nigeria following a trip to the country. The virus was later identified as being a member of the west African clade of Mpox virus [36].

### 5.6. Current Outbreak (2022)

Mpox was confirmed as a continuing outbreak in May of 2022, beginning with a cluster of cases reported from the UK [36]. The first confirmed case of the disease was identified on 6 May 2022 and occurred in a traveller from Nigeria (where it is endemic); however, there is some evidence that cases had already been spreading in Europe during the preceding months [50]. A steadily growing number of cases have been reported from an increasing number of countries and regions since 18 May 2018, most notably from North and South America, Asia, Africa, and Australia [51]. As of 9 June, 1290 new cases have been confirmed [52].

## 6. Clinical Features

**Figure 3:**
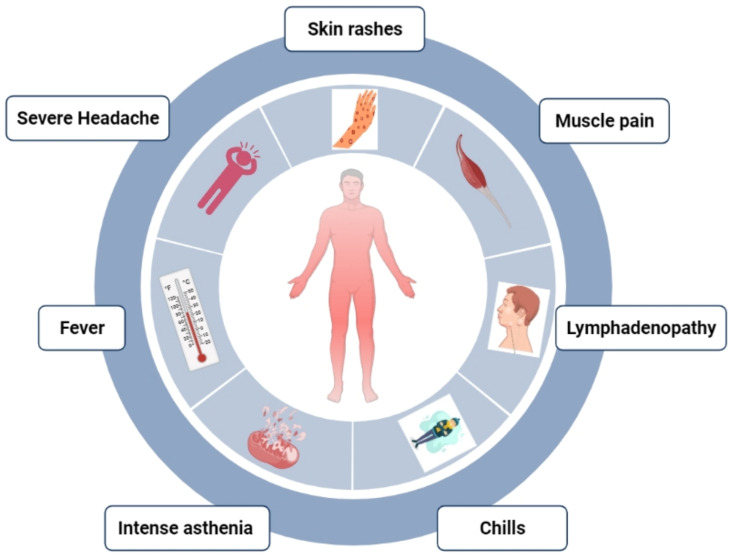
Frequently occurring symptoms of MPXV in humans.

Mpox has an incubation period (the period of time from the infection to the occurrence of symptoms) that is usually between six and thirteen days but could be anywhere from five to twenty [53]. The duration of the infection can be split into two periods. The initial phase of the infection (lasts between 0 and 5 days) is characterized by fever, severe headache, lymphadenopathy (swelling of the lymph nodes), back pain, myalgia (muscle pain) and intense asthenia (lack of energy). Lymphadenopathy is a distinctive feature of Mpox compared to other diseases that may initially appear similar (chickenpox, measles and smallpox) (Figure 3) [54]. An eruption of the skin usually begins within one to three days after the appearance of fever. It is more common for the rash to appear on the face and extremities rather than on the trunk when the condition is present. In 95% of cases, the face and palms of the hands are affected, whereas it only affects 75% of cases on the soles of the feet. Aside from the oral mucous membranes (in 70% of cases), other types of mucous membrane, genitalia (30%), conjunctivas (20%), and corneas (20%), are also affected [55]. Generally, the lesions begin as macules (lesions with a flat base) and then progress into papules (lesions that are slightly raised and hard), vesicles (lesion filled with clear fluid), pustules (lesions filled with yellowish fluid), and crusts, which eventually dry up and fall off. In general, there are between a few and several thousand lesions present. Lesions can coalesce and slough off in large sections when they are severe enough [37].

An infection of Mpox usually lasts from two to four weeks, with symptoms lasting from the start of the disease to the end. Infection severity is more common in children and is dependent on the level of virus exposure, the patient’s health status, and the type of complications. The severity of the infection may be affected by underlying immunodeficiencies [56]. Owing to the cessation of smallpox vaccination campaigns globally after the eradication of the disease, persons younger than 40 to 50 years of age (depending on the country) may be more susceptible to Mpox than in the past because of their immune systems being ineffective against smallpox. In addition, Mpox can also cause secondary infections such as bronchopneumonia, sepsis, encephalitis and corneal infection with ensuing loss of vision. The extent to which infections may occur asymptomatically is not known [57]. During the outbreak of Mpox in 2022, many of the patients presented with genital and peri-anal lesions, fever, swollen lymph nodes, and pain when swallowing [58], with some of the patients manifesting only a few sores as a result of the disease [59].

In the general population, the case fatality ratio for Mpox historically ranges from 0 to 11%, while young children’s case fatality rates tend to be higher. Currently, it is estimated that the case fatality ratio is between 3 and 6% [57].

## 7. Diagnosis

Among other possible diagnoses that must be considered when making a clinical differential diagnosis are chickenpox, measles, bacterial skin infections, scabies, syphilis and medication-associated allergies. Lymphadenopathy is a clinical feature that assists in distinguishing Mpox from chickenpox or smallpox at the prodromal stage of the illness [60]. If Mpox is suspected, a sample should be collected and safely transported to a laboratory with the necessary capabilities. In order to confirm the presence of Mpox, skin lesion material including swabs of the lesion surface and/or exudate, roofs from multiple lesions, or crusts from skin lesions, are the recommended specimen type for laboratory testing via conventional polymerase chain reaction (PCR), real-time PCR, or PCR in combination with DNA sequencing. Therefore, specimens should be packaged according to national and international requirements and should be shipped accordingly [61].

The traditional techniques of viral isolation and electron microscopy, as well as immunohistochemistry, remain valid, but require advanced technical skills and a sophisticated laboratory. Specimens can be examined using real-time polymerase chain reaction (PCR) to detect orthopoxvirus and Mpox virus. These methods are sensitive and can detect viral DNA efficiently. Presently, real-time PCR works best in a major laboratory, which limits its application as a real-time diagnostic tool in rural areas with limited resources [62]. The use of real-time PCR for diagnostic purposes outside of major laboratories may become more feasible due to advances in technology. Antibody-based diagnostics can be used to determine the cause of cases identified retrospectively. Orthopoxvirus immunological assays are cross-reactive with a variety of orthopoxviruses, making them useful in areas where the virus responsible for the illness is already known [63]. For retrospective patients who have been exposed to orthopoxviruses, including through vaccination, anti-orthopoxvirus antibody G (IgG), alone, is not enough to diagnose the disease. However, serological assays assessing anti-orthopoxvirus immunoglobulin M (IgM) are more useful for diagnosing recent retrospective infections, including in individuals who have previously been vaccinated [64].

The most appropriate samples for diagnosing Mpox are from the skin lesions—the roof and the fluid from vesicles and pustules, and the crusts of the skin lesions. Biopsies can also be performed under certain circumstances. It is essential to store lesion samples in a sterile dry tube (no viral transport media is necessary) and to keep them cold. A PCR test is usually inconclusive since viremia tends to last only for a short time relative to the time the specimens are collected after symptoms begin, and specimens cannot be collected routinely from patients [65].

There has been little progress in developing a point-of-care test that can be deployed in the field. Based on lesion samples from acute infections caused by orthopoxvirus, a recent pilot study of the Tetracore Orthopox Bio Threat Alert showed promising results. This assay has shown that vaccinia and Mpox viruses can be reliably detected in serum preparations containing 107 plaque-forming units/mL; five out of six clinical specimens were correctly identified [60]. Despite not being specific to the Mpox virus, this assay could be used for orthopoxvirus confirmation by proxy in Mpox-endemic areas, and it is critical to test this in endemic settings. Currently, the WHO recommend nucleic acid amplification testing (NAAT) to detect the Mpox viral genome, hemagglutinin, the acidophilic-type inclusion body and the crmB genes [66]. However, there is still a need for developing assays that can be tested in very basic environments with limited training of personnel, since patients with Mpox virus often seek diagnosis and care at rural clinics and hospitals without electricity. Specific information is critical to allow for an accurate interpretation of test results such as the date of the onset of fever, the date of the onset of rash, the date of specimen collection, the current state of the individual’s condition (stage of rash), and the patient’s age [65].

## 8. Therapeutics and Vaccines

Clinical care for Mpox should be fully optimized to alleviate symptoms, manage complications, and prevent long-term sequelae. Patients should be offered fluids and food to maintain adequate nutritional status and treated for any secondary bacterial infections, which can lead to a high mortality rate, especially in critically ill patients [67,68]. During outbreaks, several treatments are used to tackle these infections. For example, an antiviral agent known as tecovirimat, or TPOXX, ST-246, is FDA approved for smallpox infections. It is currently used for other orthopoxvirus infections, including Mpox-infected patients in animal and human studies, but it is still not FDA approved for clinical use. The use of tecovirimat should be monitored in a clinical research context with prospective data collection [55]. The information about a few drugs that can be utilized for the treatment of Mpox infection are presented here.

### 8.1. Tecovirimat (TPOXX, ST-246)

Tecovirimat is an antiviral drug that has been approved by the United States Food and Drug Administration (FDA) to treat smallpox infection in adults, as well as children [69]. It is recommended to take antiviral medication for those who have severe illness, or those who are at risk of developing it, or who have lesions in their eyes, mouth, or anogenital region that may signal the presence of the disease. Although there are no clinical data available at this time regarding tecovirimat, it appears to be well-tolerated and has the potential of decreasing the duration of an infection and the shedding of viral particles. There is currently a clinical trial (STOMP) being carried out to assess how well tecovirimat treats Mpox [70]. The Center for Disease Control and Prevention (CDC) recommends taking the medication tecovirimat for pregnant or nursing women who have been infected with Mpox virus. In spite of this, the only source of information regarding the effects of tecovirimat on the foetus comes from animal studies [71]. In those studies, tecovirimat was administered orally to animals at doses approximately 23 times higher than those that are generally recommended for humans in the form of a prescription drug. However, no specific adverse effects on the foetus were observed during the study [72].

There are many children with Mpox that need to be treated with tecovirimat if they have severe disease, such as airway obstruction, confluent lesions, and encephalitis. In addition, tecovirimat treatment may be needed for those that have consequences (cellulitis/abscess, ocular lesions, pneumonia or sepsis), as well as lesions involving anatomical sites that would cause scarring or strictures (e.g., infections of the eyes, face, or genitals). The use of antiviral medication should be considered an option for children under the age of eight, children suffering from conditions such as eczema and other skin disorders, as well as children with immunocompromised systems [1]. The safety experiments on about 360 volunteers were conducted as part of the approval process to determine whether tecovirimat has any adverse effects, and the results showed that its adverse effect profile was comparable to that of a placebo [73]. As far as side effects are concerned, headaches, nausea, and abdominal pain are the most commonly reported side effects. The median time to subjective benefit after commencing tecovirimat was three days after the start of the medication in a study that included 255 patients [74]. It is also effective against Mpox infection in nonhuman primates [72].

### 8.2. Cidofovir

In 2012, the FDA approved a new antiviral drug called cidofovir (Vistide). The drug is an injection-based antiviral drug that is used to treat cytomegalovirus infection (CMV) retinitis among AIDS patients [72]. However, it is not known whether cidofovir is effective in treating Mpox infection in humans. The effectiveness of cidofovir treatment, however, has been shown in some in vitro and animal studies caused by orthopoxviruses [75,76,77]. When a patient suffers from a severe Mpox infection, cidofovir may be considered as a possible treatment option, although it is unknown whether the treatment offers any benefits. It has been reported that the use of this drug can have significant adverse effects, such as nephrotoxicity [72].

### 8.3. Brincidofovir (CMX001 or Tembexa)

Brincidofovir is a formulation of the drug cidofovir that is taken orally. Brincidofovir may have a better safety profile than that of cidofovir. It should be noted that when treating cytomegalovirus infections with brincidofovir, serious renal damage or other side effects have not been reported in comparison to the use of cidofovir. The U.S. Food and Drug Administration (FDA) approved the use of brincidofovir for the treatment of smallpox, starting in June 2021 [78]. There have only been a few studies that have documented its effectiveness in treating Mpox infection. Based on animal studies, brincidofovir has been shown to be effective against orthopoxvirus infections [79,80,81]. However, it was documented that three Mpox patients who were given brincidofovir (200 mg orally once weekly), all experienced an increase in liver enzyme levels, resulting in the discontinuation of the treatment [82]. Although there is a need to develop strategies to use these drugs in endemic areas to treat disease [83]; nevertheless, natural products and their extracts could be a promising source for potential antiviral drugs [84,85].

The effectiveness of smallpox vaccination in preventing Mpox has been demonstrated to be about 85% in several observational studies. Therefore, previous smallpox vaccination could have a mild effect on Mpox infection. In most cases, a scar can be found on the upper arm of a person who has previously been vaccinated against smallpox. It should be noted that, at this point in time, the original (first-generation) smallpox vaccines are no longer available to the general public. There is a possibility that some laboratory technicians or health care workers may have received a recent smallpox vaccine to protect them against orthopoxviruses acquired on the job as a result of occupational exposure [86]. The FDA approved a second vaccine based on a modified attenuated version of the vaccinia virus (Ankara strain) for use in 2019 as a preventative management tool for Mpox. This vaccine consists of two doses and remains limited in availability. In order to develop smallpox and Mpox vaccines, formulations based on the vaccinia virus are utilized due to the cross-protection afforded by the antiviral response to orthopoxviruses [87,88,89].

### 8.4. Potential Therapeutic Targets

A previous study has shown that the Mpox genome shares 96.3% of its DNA with the smallpox genome, which encodes several essential enzymes and proteins that are important to its survival [90]. Consequently, the inhibition of such a broad range of enzymes and proteins through a drug molecule could represent one of the potential therapeutic strategies. A number of important potential therapeutic targets for treating Mpox infection are discussed here.

### 8.5. Thymidylate Kinase

As previously reported, thymidylate kinase has been found to form complexes with thymidine diphosphate [91]. This is a novel target of interest due to the fact that no known drugs have yet been developed to target this enzyme. It has been found that A48R plays an important role in the conversion of thymidine monophosphate into its diphosphate, as well as in the conversion of the analogues of 5′ halogenated deoxyuridine monophosphate into its diphosphate [92]. Since the active site of human thymidylate kinase exhibits substantial structural differences from the structurally related human analogue [91], it might become an attractive target for the development of thymidine analogues without being too concerned with restricting the function of the human analogue.

### 8.6. DNA Ligase

DNA ligase is an important enzyme, which is essential for virus replication. A mutation at its N-terminus has resulted in instances of resistance to antiviral drugs in this protein. This location of resistance mutations indicates that the active site of the drug is situated at this location [93], which makes it a valuable target for drug discovery.

### 8.7. D13L (Protein Trimer Complex)

A major capsid protein, D13L, is part of a protein trimer complex and contributes to the rigidity of the membrane of the virus particles; thus is particularly important in the morphogenesis of the viral particle [94]. There is a strong correlation between rifampin and the D13L protein in the vaccinia virus, which has previously been demonstrated to have an affinity for this protein [92]. As a result of its ability to bind to the D13L trimer complex, rifampin has been demonstrated to inhibit poxvirus assembly, an effect independent of its antibacterial activity [95].

### 8.8. F13L (Major Envelope Protein)

A drug called tecovirimat (formerly known as ST-246) is the only drug approved to treat poxviruses that target the F13L gene. As a major envelope protein, as well as a palmitoylated membrane protein, it plays a crucial role in the formation of extracellular envelope viruses (EEVs) as well as the entry of viruses into cells [96].

### 8.9. I7L (Cysteine Proteinase)

There is a proteinase known as I7L core proteinase that is a cysteine proteinase which cleaves the major structural and membrane proteins of both viruses and bacteria [97]. As a result of their essential role in the replication of viruses by cleaving precursor polyproteins, proteases are ideal therapeutic targets [98], and protease inhibitors for other viruses, such as HIV, have also been confirmed to be effective against other viral proteases. Due to this, Mpox virus proteases are also attractive targets for inhibiting the replication of the virus. Previously, TTP-6171 was identified as an inhibitor of the I7L enzyme in previous studies. There is also the possibility that drug-resistant I7L can be generated with mutations on the I7L gene [97].

## 9. Prevention

In order to prevent Mpox, the main prevention strategy is to raise awareness about the risk factors, and to educate people about the steps they can take to reduce their exposure to the virus. Scientists are currently studying whether and to what extent vaccination would be effective in the prevention and treatment of Mpox in many countries around the globe. As a result, certain countries have policies in place, or are developing policies, to provide vaccines to persons who may be at risk, such as laboratory personnel, rapid response teams, and healthcare workers [99].

As a means of preventing Mpox, it is necessary to reduce the risk of the disease spreading from human to human. In order to contain an outbreak, it is critical to conduct surveillance, and to identify new cases as soon as possible. Close contact with a person who is infected with the human Mpox virus is the most significant risk factor for the infection. The risk of infection is higher among health workers and household members. Thus, it is recommended that health care workers who are caring for patients or handling specimens of Mpox should follow standard infection control precautions. Whenever possible, persons who have previously been vaccinated against smallpox should be selected to be caregivers for the patient [100].

The handling of Mpox virus-infected people and animals should be carried out by trained personnel who work in laboratories that are appropriately equipped. In accordance with the WHO guidelines for the transportation of infectious substances, patient specimens must be carefully prepared for transport with three layers of packaging [101]. There are clusters of Mpox cases identified in May 2022 in several non-endemic countries with no direct travel ties to endemic areas. This is highly unusual. In order to determine the likely source of infection and limit further spread of the disease, more investigations are in progress. In addition to investigating the source of this outbreak, it is also important to examine all possible means of transmission in order to protect public health [9].

In addition, it is important to reduce the risk of transmission of zoonotic diseases. Since the advent of animal-to-human transmissions, most human infections have been transmitted through animals. In order to maintain safety, unprotected touching of wild animals, especially when they are sick or dead, as well as handling or consuming their meat, blood and other parts, must be avoided. Furthermore, all foods containing animal meat or animal parts must be thoroughly cooked before consumption [100].

Moreover, Mpox can also be reduced by limiting the trade in animals, which is another essential step toward the prevention of this disease. In a few countries in the world, legislation has been enacted which imposes restrictions on rodent imports as well as on imports of non-human primates. If captive animals are suspected of being infected with Mpox, they should be quarantined from other animals immediately and kept in isolation. It is important to quarantine any animals that might have come into contact with an infected animal, handle them in accordance with the standard precautions, and monitor them for Mpox symptoms for at least 30 days after such contact [56].

## 10. Research Challenges with Mpox

In order to better understand the dynamics of Mpox transmission and control, operational research is currently facing challenges, such as insufficient resources for detailed case investigations and contact follow-up in affected communities. A lack of adequate diagnostic facilities in the laboratory is a serious problem. Owing to the lack of laboratory diagnosis capacity and access, as well as the difficulty of diagnosing Mpox, it is difficult to discover any underlying aetiology. A seroprevalence study would help to understand the epidemiology and subclinical infection among contacts in communities [102]. The currently available serological assays are generic orthopox tests, meaning they do not specifically test for the Mpox virus. This is due to the fact that there is cross-reactivity between Mpox and smallpox viruses and, therefore, we cannot distinguish between a Mpox virus infection and prior smallpox vaccinations or other orthopoxvirus infections. In addition, these assays are not currently available in the marketplace. It has been found that, according to data collected from Nigeria, approximately 20% of 70 Mpox-negative patients with a rash illness with similar antigens also had orthopox antibodies. In order to identify other orthopoxviruses being transmitted in human and animal populations, further research, including using molecular and genomic approaches, is needed [54].

## 11. Conclusions

In endemic and non-endemic countries alike, this outbreak of Mpox has challenged economic, medical, and public health infrastructures all around the world. The impact of the virus on our lives will only be known in the future. Moreover, zoonotic viruses and pathogens are likely to continue causing outbreaks of disease in the future. As a result, in addition to curbing this outbreak, efforts must focus on ensuring that comprehensive measures are devised to prevent future outbreaks of zoonotic origin.

## Data Availability

All data generated or analysed during this study are included in this article.
